# 1750. Factors associated with respiratory pathogen panel utilization in children hospitalized with acute respiratory illness — New Vaccine Surveillance Network, Kansas City, 2017–2021

**DOI:** 10.1093/ofid/ofad500.1581

**Published:** 2023-11-27

**Authors:** Edward Lyon, Brian R Lee, Benjamin R Clopper, Heidi L Moline, Rangaraj Selvarangan, Jennifer E Schuster

**Affiliations:** Children's Mercy Hospital, Kansas City, Missouri; Children's Mercy Kansas City, Kansas City, Missouri; US Centers for Disease Control & Prevention, Buffalo, New York; Centers for Disease Control and Prevention, Atlanta, Georgia; Children’s Mercy Kansas City, Kansas City, Missouri; Children’s Mercy Kansas City, Kansas City, Missouri

## Abstract

**Background:**

Respiratory pathogen panels (RPP) are multiplex PCR platforms that detect several respiratory viruses from one specimen. For most children hospitalized with acute respiratory illness (ARI), management is supportive, and detection of a specific virus from RPP does not impact clinical care. Therefore, clinical RPP use is not standardized, and ordering is at the discretion of the clinician. We sought to understand factors associated with RPP utilization among pediatric patients hospitalized with ARI.

**Methods:**

From October 2017 to September 2021, participants < 18 years hospitalized with ARI were enrolled at a single site in the New Vaccine Surveillance Network (NVSN). Eligible patients were residents of Jackson County, MO, had one or more ARI symptoms (e.g., cough, fever, nasal congestion) lasting < 14 days, and were enrolled within 48 hours of admission. Parent interviews and medical chart reviews were conducted. All participants had a research RPP, but results were not available to the clinical providers. Clinical providers were able to order a clinical RPP (cRPP), for which they received test results. Characteristics of NVSN enrollees hospitalized with ARI with and without a cRPP are described. Lastly, medical complexity was assessed via the pediatric complex chronic conditions classification system (CCC) then analyzed via chi- square test between groups.

**Results:**

During the study period, 1,038 participants were enrolled, and 555 (53.4%) received a cRPP. Most, 299 (53%), cRPPs were ordered in the emergency department or urgent care before admission. Age was a significant factor associated with cRPP use (Table 1). cRPP participants were more likely to have complex chronic conditions, and/or technology dependence. No difference in cRPP use was observed by race/ethnicity, payer status, or sex. More participants were enrolled in 2020-2021, but the overall usage of cRPP is similar between years (Table 2).

**Figure:**
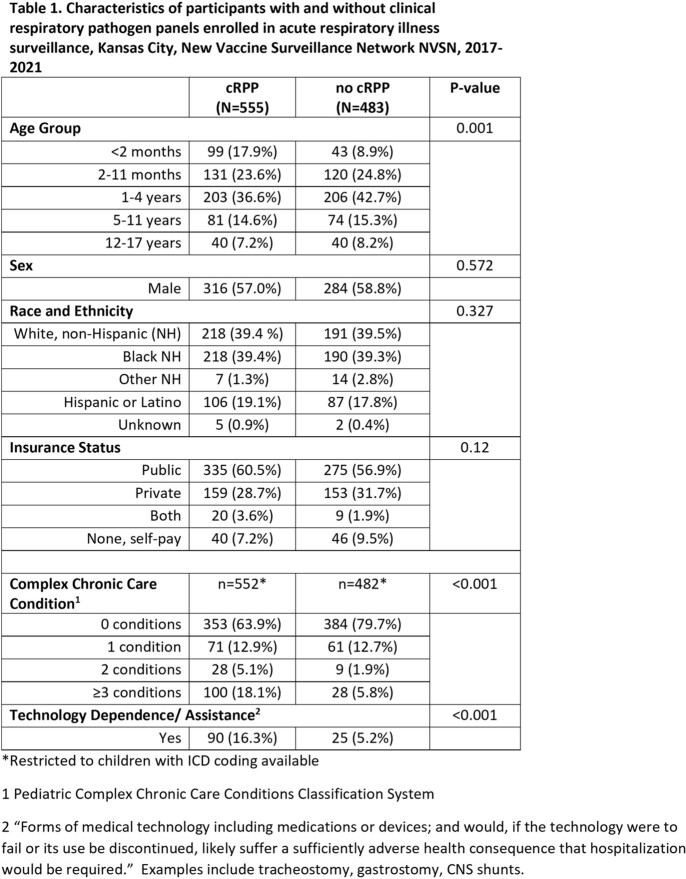

**Figure:**
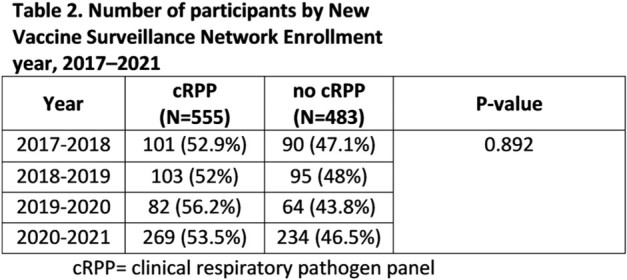

**Conclusion:**

In this large cohort of children hospitalized with ARI, medical complexity, technological dependence, and age < 2 months were associated with increased utilization of cRPPs. Understanding the impact of cRPP on clinical care requires further investigation to better understand the utility of these tests.

**Disclosures:**

**Rangaraj Selvarangan, BVSc, PhD, D(ABMM), FIDSA, FAAM**, Abbott: Honoraria|Altona Diagnostics: Grant/Research Support|Baebies Inc: Advisor/Consultant|BioMerieux: Advisor/Consultant|BioMerieux: Grant/Research Support|Bio-Rad: Grant/Research Support|Cepheid: Grant/Research Support|GSK: Advisor/Consultant|Hologic: Grant/Research Support|Lab Simply: Advisor/Consultant|Luminex: Grant/Research Support

